# The Pronator Teres Muscle Revisited: Morphological Classification, Neurovascular Entrapment, and Surgical Implications

**DOI:** 10.3390/jcm14217474

**Published:** 2025-10-22

**Authors:** Marta Podlasińska, Ingrid C. Landfald, Zuzanna Adamczyk, Bartłomiej Szewczyk, Łukasz Olewnik

**Affiliations:** 1 Department of Clinical Anatomy, Mazovian Academy in Płock, 09-402 Płock, Poland; 2 VARIA Research Laboratory, Department of Clinical Anatomy, Mazovian Academy in Płock, 09-402 Płock, Poland; 3 VARIANTIS Research Laboratory, Department of Clinical Anatomy, Mazovian Academy in Płock, 09-402 Płock, Poland; 4 Department of Anatomical Dissection and Plastination, Mazovian Academy in Płock, 09-402 Płock, Poland; b.szewczyk@mazowiecka.edu.pl

**Keywords:** pronator teres muscle, pronator syndrome, median nerve entrapment, forearm anatomy, ultrasonography, magnetic resonance imaging

## Abstract

**Background:** The pronator teres (PT) muscle, although traditionally described as a constant two-headed forearm flexor, demonstrates considerable morphological variability. These variants play a crucial role in the pathogenesis of pronator syndrome, a rare but clinically significant entrapment of the median nerve in the proximal forearm. Despite growing interest, there is no widely adopted classification integrating anatomy, imaging, and surgical relevance. This review summarises and contextualises current classifications of the pronator teres in relation to median nerve entrapment, with emphasis on their anatomical, imaging, and surgical relevance. **Methods:** We performed a narrative review of the literature using PubMed, Scopus, and Web of Science (1960–2025). Studies were included if they reported cadaveric findings, imaging features, or clinical management of PT-related median nerve entrapment. Representative anatomical and clinical sources were analysed to synthesise a morphology-based framework. **Results:** We identified three morphological types of the PT: Type I (median nerve between humeral and ulnar heads, 74%), Type II (beneath both heads, 12%), and Type III (beneath the humeral head only, 14%). Each type demonstrates distinct entrapment mechanisms and imaging features. Dynamic ultrasound and advanced MRI sequences, particularly MR neurography, have been reported to improve diagnostic confidence but remain underutilised. Published reports describe differing management approaches by type, with variable outcomes. Tables and summary boxes compile previously published findings on entrapment potential, imaging pitfalls, and surgical approaches by type. **Conclusions:** This review summarises existing classifications linking PT variability to median nerve entrapment. Such integration may have potential clinical relevance but requires further empirical validation. Future studies should standardise imaging protocols, validate electrodiagnostic correlations, and explore functional classifications incorporating clinical, radiological, and anatomical data.

## 1. Introduction

### 1.1. General Anatomy and Function of the Pronator Teres

The pronator teres (PT) muscle is a superficial flexor of the forearm, typically consisting of two heads: the humeral head originating from the medial epicondyle of the humerus and the ulnar head arising from the coronoid process of the ulna. Its insertion is located on the lateral surface of the radius, where it exerts its primary action of pronation of the forearm, in synergy with the pronator quadratus. In addition, the PT contributes to weak flexion of the elbow joint [1,2].

### 1.2. Functional Role and Clinical Relevance in Median Nerve Compression

Clinicians recognise the PT as a potential site of median nerve compression, leading to the clinical entity known as pronator syndrome [3,4]. Patients typically present with pain in the proximal forearm, weakness during pronation against resistance, and sensory disturbances in the distribution of the median nerve. Differentiating pronator syndrome from carpal tunnel syndrome or anterior interosseous nerve neuropathy is crucial in clinical practice, given the overlapping but distinct neurological presentations. The literature emphasises careful differentiation between pronator syndrome, carpal tunnel syndrome, and anterior interosseous neuropathy in suspected cases. Pronator syndrome remains relatively uncommon and often under-recognised in clinical practice, contributing to diagnostic delay and misclassification as distal neuropathies [1,3,4,5].

### 1.3. The Need for an Updated Morphological Classification

Although early anatomical descriptions portrayed the PT as a relatively constant muscle, subsequent investigations have demonstrated considerable variability in its morphology. Reported differences include supernumerary heads, accessory muscular slips, and diverse spatial relationships with the median nerve [6,7]. Such anatomical heterogeneity is clinically significant, since aberrant or hypertrophic muscle fibres may serve as predisposing factors for nerve entrapment. Recognising this variability, Olewnik et al. [8] proposed a comprehensive morphological classification of the PT based on cadaveric dissections in a Central European population. This system integrates the spectrum of anatomical variants with their potential to compress the median nerve, thereby providing a practical framework for both diagnostic imaging and surgical decision-making. This classification-based framework has been interpreted in the literature as a model for understanding entrapment risk and guiding anatomical interpretation. Therefore, this review summarises how existing classifications of the pronator teres have been interpreted across clinical, radiological, and surgical literature in relation to median nerve entrapment. Despite numerous anatomical descriptions, no widely adopted scheme explicitly links variant morphology to imaging findings and procedural descriptions in published literature. Accordingly, this review provides a descriptive synthesis linking existing morphological classifications of the pronator teres to published clinical, radiological, and surgical observations; it does not propose a validated diagnostic or management system and generalisability beyond the source cadaveric cohort remains uncertain.

## 2. Materials and Methods

This work is a narrative (non-systematic) review. A structured literature review was performed to identify anatomical and clinical studies concerning the PT muscle and pronator syndrome, covering foundational anatomical variability and clinical presentations [1,4,9,10]. The databases PubMed, Scopus, and Web of Science were searched for articles published between 1960 and 2025, using combinations of the following keywords: “pronator teres,” “pronator syndrome,” “median nerve entrapment,” and “proximal forearm neuropathy.” [1,4,9,10]. The databases PubMed, Scopus, and Web of Science were searched for articles published between 1960 and 2025, using combinations of the following keywords: “pronator teres,” “pronator syndrome,” “median nerve entrapment,” and “proximal forearm neuropathy.”

After screening, 28 full-text studies met the inclusion criteria and were synthesised, comprising six cadaveric/anatomical, ten imaging/electrodiagnostic, and twelve clinical/surgical reports. Additional narrative reviews and methodological overviews were consulted for context but were not counted among the included studies. As a narrative review, no PRISMA checklist, formal risk-of-bias assessment, or quantitative evidence grading was performed; selection and interpretation bias are acknowledged limitations of this design.

### Inclusion Criteria Comprised the Following

Cadaveric studies documenting morphology, morphometry, or variability of the PT and its relation to the median nerve [4,6,10,11,12];

Imaging studies employing MRI or ultrasonography (US) to evaluate the PT region or demonstrate features consistent with proximal median nerve entrapment [13];

Clinical case reports/series and surgical studies addressing pronator syndrome, including indications, techniques, and outcomes of decompression [1,7,9,14,15].

Search strategy. PubMed (last search: 6 October 2025):

((“pronator teres”[Title/Abstract] OR “Pronator Teres”[MeSH] OR “pronator syndrome”[Title/Abstract]) AND (“median nerve”[Title/Abstract] OR “Median Nerve”[MeSH]) AND (entrapment OR compression OR neuropathy)); Filters: Humans; English; 1960/01/01–2025/10/06.

Scopus (last search: 6 October 2025; TITLE-ABS-KEY):

(“pronator teres” OR “pronator syndrome”) AND (“median nerve”) AND (entrapment OR compression OR neuropathy); Limits: English; 1960–2025.

Web of Science (last search: 6 October 2025; TS):

((“pronator teres” OR “pronator syndrome”) AND (“median nerve”) AND (entrapment OR compression OR neuropathy)); Timespan: 1960–2025; Language: English; Document types: Article, Review.

Where database-specific syntax differed, the same Boolean logic was mirrored.

To minimise selection bias, titles and abstracts were independently screened by two reviewers, data extraction was cross-checked, and disagreements were resolved by consensus with the senior author. No language restriction was applied at the search stage, but only studies available in English were included in the final analysis. Reference lists of eligible articles were screened to identify additional relevant sources. Given the narrative design, a risk of selection and interpretation bias remains inherent despite these mitigation steps.

Study selection: Identification: records retrieved from PubMed, Scopus, and Web of Science within 1960–2025 (Humans; English). Screening: titles/abstracts independently screened by two reviewers (M.P., I.L.); disagreements resolved by consensus with the senior author (Ł.O.). Eligibility: full texts assessed against predefined inclusion criteria (cadaveric/anatomical; imaging; clinical/surgical relevance to PT-related median neuropathy). Included: 28 studies were synthesised.

For each included paper, data were extracted on study type (cadaveric, imaging, clinical), PT–median nerve relationships, diagnostic modalities (US/MRI/EMG), and management/outcomes where applicable. When overlapping cohorts were identified, the most comprehensive dataset was retained; morphological descriptors were harmonised to a three-type framework.

Results of the Review. Twenty-eight full-text studies were included (approximately six cadaveric/anatomical, ten imaging/electrodiagnostic and twelve clinical/surgical). No new cadaveric dissections or original imaging cohorts were generated for this review. The three-type framework used here was derived from Olewnik et al. [8] and subsequently mapped against imaging and surgical reports to facilitate type-specific interpretation. Sections on imaging, clinical implications and surgical access present an interpretive synthesis (Discussion) of these results.

The three-type framework used here is derived from Olewnik et al. [8] and is applied solely as an interpretive scaffold to map published findings from anatomical, imaging, and surgical studies. Prevalence figures quoted in the text reflect previously published cadaveric data rather than new estimates.

## 3. Morphological Classification of the Pronator Teres Muscle

**Figure 1:**
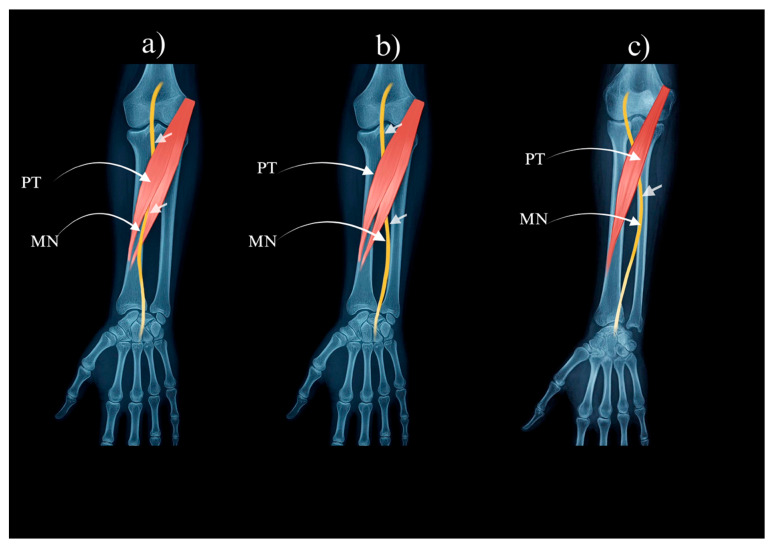
Pronator teres muscle types: (**a**) Type I of the pronator teres. (**b**) Type II of the pronator teres. (**c**) Type III of the pronator teres. PT—pronator teres. MN—median nerve.

Olewnik et al. [8] proposed a three-type classification system of the PT muscle, based on cadaveric dissection of a Central European cohort. In this review, the framework is used as an interpretive scaffold to summarise previously published observations; it is not presented as a validated or universally applicable diagnostic system, and generalisability beyond the source cohort remains uncertain. This classification integrates the presence or absence of the ulnar head and the anatomical relationship of the median nerve with the PT heads:

Type I: Both humeral and ulnar heads are present; the median nerve passes between them (reported prevalence in the source cohort ≈ 74%)—Figure 1a.

Type II: Both heads are present; the median nerve courses deep to both heads (≈12% in the source cohort)—Figure 1b.

Type III: The ulnar head is absent; the median nerve passes beneath the humeral head (≈14% in the source cohort)—Figure 1c.

Prevalence figures are drawn from the original cadaveric dataset (Olewnik et al. [8]) and are included here descriptively; no new estimates were generated.

This framework demonstrates that although the PT is generally described as a two-headed muscle, notable anatomical variation exists. The course of the median nerve in relation to the PT muscle bellies has been reported to vary across individuals and is clinically relevant, as it may influence the likelihood and site of neurovascular entrapment. To facilitate interpretation, the classification reported by Olewnik et al. [8] is summarised in Table 1. This overview compiles previously published descriptions of PT variability and their reported associations with potential neurovascular entrapment. Alternative descriptions in the literature emphasise accessory slips and head configurations, which partly overlap with the arrangement of Types II and III described by Olewnik et al. [8], as well as by other authors [7,10,11]. Divergences across schemas largely reflect whether variants are defined by the presence or number of heads and accessory slips versus the spatial relationship to the median nerve [8]. In this review, emphasis is placed on the latter approach, as it has been considered in the literature to most directly inform imaging interpretation and surgical planning.

## 4. Neurovascular Entrapment Potential by Type

### 4.1. Type I—Between the Two Heads

Type I, in which the median nerve passes between the humeral and ulnar heads of the pronator teres, has been reported as the most common configuration associated with neurovascular entrapment [8]. Compression has been described when both heads are hypertrophic or when fibrous bands traverse the intermuscular space, narrowing the passage for the nerve [1,2,4,16]. Clinically, this configuration corresponds to the classical presentation of pronator syndrome described in the literature, often characterised by proximal forearm pain, weakness during resisted pronation, and paraesthesia in the distribution of the median nerve [3]. A recurring diagnostic challenge noted in published reports is distinguishing this entity from carpal tunnel syndrome, as both may produce overlapping sensory symptoms in the hand. However, the presence of proximal forearm pain, tenderness over the pronator region, and symptom exacerbation with resisted pronation have been reported as helpful discriminators [17]. The literature emphasises differentiation of pronator syndrome from carpal tunnel and anterior interosseous neuropathies. While carpal tunnel syndrome presents with nocturnal paraesthesia and distal hand symptoms, and anterior interosseous neuropathy primarily causes motor deficits without sensory loss, pronator syndrome typically includes proximal forearm pain and sensory disturbances extending into the palm.

### 4.2. Type II—Beneath Both Heads

In Type II, the median nerve courses deep to both the humeral and ulnar heads of the PT, creating a narrow tunnel with muscular boundaries both superiorly and inferiorly [8]. This arrangement has been described as conferring a relatively higher entrapment potential than Type I, given the double-headed tunnel and increased susceptibility to compression (see Section 4.1 for mechanistic details) [2,9]. Clinically, reports indicate more pronounced motor findings, including selective weakness of pronation while other median-innervated forearm muscles retain normal function. This presentation has been noted to reflect the focal nature of entrapment at this level and may assist in distinguishing Type II compression from distal neuropathies such as anterior interosseous nerve palsy or carpal tunnel syndrome [1,3].

### 4.3. Type III—Beneath the Humeral Head Only

Type III is characterised by the absence of the ulnar head, with the median nerve coursing deep to the humeral head alone [8]. Although less common than Types I and II, this configuration has been described as clinically relevant in case reports and small series. Symptoms may resemble those of classical pronator syndrome despite the absence of one muscular head [3]. Because the nerve is compressed solely by the humeral head, entrapment has been described as often subtler or more difficult to detect. Mechanistic considerations are similar to those detailed for Type I (Section 4.1); hence, awareness of this variant has been highlighted in the literature as useful for preoperative planning in minimally invasive or targeted decompression approaches [9,10,18].

The reported entrapment potential of each morphological type is summarised in Table 2, with descriptive observations compiled in Table 3.

## 5. Imaging Features and Diagnostic Pitfalls by Type

Recent studies have highlighted complementary roles of dynamic ultrasound (US) and magnetic resonance (MR) neurography for assessing median neuropathies, with measurement criteria that may be transferable to proximal entrapments [19,20].

### 5.1. Type I—Between the Two Heads

In Type I, US has been reported to demonstrate the MN traversing the intermuscular cleft between the HH and UH of the PT. High-resolution US can visualise calibre changes or dynamic compression during resisted pronation [13,21]. Dynamic US may also show tethering or abnormal MN excursion during pronation–supination, findings not always visible on static imaging.

MRI, particularly T2-weighted and fat-suppressed sequences, has been shown to depict focal thickening or hyperintensity of the MN at the presumed entrapment site, with perineural oedema [7]. Axial sections are generally considered most informative for delineating the MN within the intermuscular tunnel, whereas coronal images may assist in assessing longitudinal continuity.

A commonly reported diagnostic pitfall is misinterpreting proximal Type I entrapment as distal CTS when imaging is confined to the wrist. Isolated distal evaluation may therefore miss the proximal compression site, potentially delaying recognition [3]. When proximal entrapment is suspected, several reports have indicated that combining MRN with dynamic US can improve detection and reduce interpretive ambiguity [19].

### 5.2. Type II—Beneath Both Heads

In Type II, the MN runs deep to both the HH and UH, which has been reported to limit US visualisation. The deeper location reduces the acoustic window, and overlying muscular bulk may obscure the compression site. Dynamic US performed during resisted pronation has been described as increasing diagnostic sensitivity, though findings can remain subtle [13,22]. Colour Doppler has occasionally been used to differentiate vascular from fibrous structures.

MRI in this configuration often yields nonspecific findings. Although T2 hyperintensity or calibre change may be observed, these features are frequently indeterminate and can mimic distal neuropathies. Coronal and sagittal planes can help confirm MN continuity, but axial images are generally regarded as most reliable [23,24]. Advanced modalities such as MRN or diffusion tensor imaging (DTI) have been proposed to improve detection, although their clinical availability remains limited.

Type II has therefore been characterised in the literature as diagnostically challenging, as entrapment beneath both heads may be poorly demonstrated on static imaging alone. Dynamic US combined with provocative manoeuvres and EDX testing has been suggested to increase diagnostic confidence [4,9].

### 5.3. Type III—Beneath the Humeral Head Only

In Type III, the absence of the UH eliminates the intermuscular cleft normally visible on US. Consequently, the MN lies exclusively beneath the HH, which may complicate identification of the compression site. High-resolution US can sometimes demonstrate calibre changes or focal narrowing, though these are generally subtler than in Types I or II [8,18].

MRI in this variant may require higher-contrast sequences to depict abnormalities. Standard T1- or T2-weighted images may not clearly show compression; therefore, STIR or MRN sequences have been reported to better reveal intraneural oedema and subtle signal changes [14]. Recognition of this configuration has been emphasised in the literature as useful for preoperative planning of minimally invasive procedures, where the absence of the UH reduces anatomical landmarks.

Although Type III is less common, awareness of this arrangement has been considered important for radiologists and clinicians evaluating unexplained proximal MN neuropathy. Mechanistic interpretations largely mirror those discussed for Type I (Section 4.1); thus, the absence of the intermuscular landmark should not be assumed to imply negligible entrapment risk [9].

The reported imaging characteristics of each morphological type and their associated diagnostic pitfalls are summarised in Table 4, with literature-based observations outlined in Table 5.

## 6. Clinical Implications and Management Strategies by Type

### 6.1. Type I—Between the Two Heads

In Type I, where the median nerve traverses the cleft between the humeral and ulnar heads, the literature generally describes conservative management such as physiotherapy, activity modification, and US-guided perineural injections as the initial approach [13,21,25]. When symptoms persist despite such measures, surgical decompression has been reported in case series and cohort studies as an option associated with favourable outcomes, provided that proximal entrapment is correctly differentiated from distal neuropathies, particularly CTS [4,9,12]. Long-term improvement has been documented when fibrous bands were completely released [10,12]. Several authors have emphasised the importance of distinguishing this variant from lacertus syndrome, as compression beneath the lacertus fibrosus can mimic PT syndrome clinically [26]. The principal management pathways for all morphological types are summarised in Table 6, and a concise overview of reported management trends is provided in Box 1.

Box 1 **Summary of reported management trends for PT-related median neuropathy.**

- Conservative care (physiotherapy, activity modification, US-guided injections) is most often described as first-line, with surgery reserved for persistent cases.
- **Type II** variants frequently require decompression of both heads with removal of fibrous bands.
- **Type III** may pose intra-operative challenges due to the absent ulnar head, emphasising the importance of detailed pre-operative imaging.
- Accurate differentiation from **carpal tunnel syndrome (CTS)** through combined clinical and imaging assessment is consistently stressed in the literature.
- Use of **dynamic ultrasound** and meticulous pre-operative planning has been reported to reduce the risk of incomplete release.

### 6.2. Type II—Beneath Both Heads

In Type II, conservative measures have been reported as less effective than in Type I. Published studies indicate that patients with this configuration more frequently undergo surgical decompression, which typically involves release of both the HH and UH and excision of accessory fibrous structures [3,8,9]. This type has been described as technically demanding and associated with a higher recurrence risk if decompression is incomplete.

### 6.3. Type III—Beneath the Humeral Head Only

Type III, characterised by the absence of the ulnar head, has been described as more likely to respond to conservative management, with surgery reported mainly in cases of persistent compression attributed to hypertrophy or fibrosis of the HH [9]. The absence of the UH as a surgical landmark has been noted to pose intraoperative challenges, highlighting the role of preoperative imaging in accurately defining variant anatomy, particularly when minimally invasive or endoscopic decompression is being considered [12].

## 7. Surgical Access and Procedural Considerations by Type

### 7.1. Type I—Between the Two Heads

In Type I, a standard anterior approach through the elbow flexor crease has been described as providing excellent exposure of both heads and the intermuscular tunnel, facilitating straightforward decompression [1,4]. This approach has been reported to allow identification of fibrous bands or hypertrophic fibres that may compress the nerve; careful inspection of the intermuscular cleft has been emphasised in the literature to minimise the risk of incomplete release [10].

### 7.2. Type II—Beneath Both Heads

In Type II, the median nerve lies deep to both the humeral and ulnar heads, often requiring an extended anterior exposure with deeper dissection [8,9]. Attention to vascular structures adjacent to the ulnar head has been highlighted, as these may increase the risk of intraoperative bleeding and add technical complexity to dissection. This variant has been described as technically demanding, with both heads typically released and accessory fibrous arches identified to help prevent recurrence [10,12]. Incomplete release at this level has been reported as a potential cause of persistent or recurrent symptoms [12].

### 7.3. Type III—Beneath the Humeral Head Only

In Type III, the absence of the ulnar head eliminates a common anatomical landmark, which has been noted to complicate intraoperative orientation. Preoperative imaging has been reported as useful to accurately localise the compression site and to guide surgical planning [7,9]. During decompression, careful dissection of the humeral head and direct visualisation of the nerve have been described as approaches to reduce iatrogenic injury [18]. This variant has been discussed as particularly relevant when minimally invasive or endoscopic techniques are considered, as the reduced number of anatomical landmarks may increase the risk of incomplete release [10].

The surgical approaches, their advantages, and main technical challenges are detailed in Table 7, with additional guidance summarised in Clinical Surgical Tips (Box 2).

Box 2 **Summary of intraoperative considerations reported in the literature, highlighting variant-specific approaches and key risk factors during decompression.**

- The surgical approach is selected according to the anatomical configuration of the pronator teres identified preoperatively.
- In Type I, complete visualisation of the intermuscular cleft and fibrous bands has been emphasised to minimise recurrence.
- Type II involves deeper dissection and proximity to vascular structures, reported as major technical challenges.
- In Type III, preoperative imaging has been noted as critical for orientation due to the absence of the ulnar head landmark.
- Minimally invasive or endoscopic techniques have been described only when variant anatomy and nerve course are clearly delineated.

## 8. Future Directions

Despite growing knowledge of PT variability and its clinical significance, current evidence remains limited, particularly for Types II and III, underscoring the need for more robust clinical–imaging correlation studies. Several key areas warrant further investigation:

### 8.1. Standardisation of Imaging Protocols

The literature highlights the need for standardised dynamic ultrasound (US) *and* magnetic resonance (MR) neurography protocols across centres. Musculoskeletal (MSK) US has been described as a rapid, accurate, and cost-efficient tool for evaluating the median nerve (MN) [27], while reviews of MR neurography emphasise the importance of harmonising advanced imaging parameters between institutions [28,29]. Proposed criteria, including cross-sectional area (CSA) thresholds, STIR/T2 features, and structured reporting checklists, require validation through prospective, multicentre studies focusing on proximal median entrapment [19,20]. Recent overviews of pronator teres syndrome also underline the importance of consistent imaging and clinical pathways [5].

### 8.2. Histopathological and Biomechanical Analyses

Further histopathological and biomechanical investigations are needed to clarify inter-type differences, particularly those related to fascial architecture, connective tissue stiffness, and local mechanical stress distribution.

### 8.3. Integration of Intraoperative Navigation

Future studies may explore the potential role of intraoperative neuronavigation in improving localisation of entrapment sites and reducing iatrogenic risk, especially in less common configurations such as Types II and III.

### 8.4. Development of Imaging–Functional Classifications

Development of integrated imaging–functional classifications has been proposed to bridge the gap between anatomical variability and clinical assessment. Combining electrodiagnostic (EDX) signatures from proximal, distal, and dual MN lesions could help refine diagnostic interpretation and management strategies [30].

Recent case-based studies report that EDX–imaging concordance supports more targeted evaluation in PT-related neuropathies [31]. For instance, the combination of MR neurography and diffusion tensor imaging (DTI) parameters—fractional anisotropy, apparent diffusion coefficient (ADC), and cross-sectional area—has improved diagnostic precision in carpal tunnel syndrome (CTS) [15]. Similarly, comparative studies of high-resolution US and MR neurography demonstrate complementary diagnostic value for MN entrapment [32].

## 9. Limitations

### 9.1. Lack of Variant-Specific Electrodiagnostic Data

There is a lack of comprehensive EDX and nerve conduction datasets differentiating findings among PT Types I–III. This limitation constrains the ability to correlate electrophysiological features with specific anatomical variants and to define variant-dependent diagnostic criteria.

### 9.2. Limited Operative Evidence for Types II and III

Evidence from surgical case series involving Types II and III remains scarce and heterogeneous. Few studies provide long-term follow-up or detailed correlation between intraoperative findings and imaging data. This gap highlights the need for prospective studies to clarify outcome variability and procedural safety in these less common variants.

### 9.3. Restricted Application of Dynamic Imaging

Dynamic US and advanced MRI techniques (e.g., MR neurography) remain underused in clinical evaluation of proximal MN entrapment. This limited adoption contributes to delayed or inaccurate diagnosis, particularly when distal neuropathies are presumed.

As this work represents a narrative review rather than a systematic synthesis, a formal risk-of-bias assessment was not conducted. Dual screening and consensus-based data harmonisation were applied to minimise interpretive bias. Nonetheless, the lack of high-quality clinical trials and imaging–surgery correlation studies—especially for Types II and III—represents a key evidence gap requiring targeted future investigation.

## 10. Conclusions

This review summarises the anatomical, imaging, and clinical relevance of pronator teres (PT) morphological variability. The three-type framework (Types I–III) offers a descriptive model for understanding mechanisms of proximal median nerve compression and their potential diagnostic implications.

Rather than prescribing management or imaging strategies, this synthesis highlights how awareness of type-specific anatomy may inform interpretation of existing literature on entrapment risk, diagnostic patterns, and surgical challenges.

The proposed framework should be regarded as interpretive rather than directive, underscoring the need for systematic imaging validation, prospective surgical correlation, and outcome-based studies to confirm its clinical applicability.

## Figures and Tables

**Table 1:** Morphological classification of the pronator teres muscle according to Olewnik et al. [8] Compiled summary of previously published cadaveric findings.

Type	Description	Median Nerve Course	Prevalence (%)	Reported Clinical Relevance
I	Both humeral and ulnar heads present	Between the two heads	74%	Classical configuration described in pronator syndrome; most frequent potential entrapment site
II	Both heads present	Deep to both heads	12%	Narrow muscular tunnel; reported higher likelihood of persistent compression
III	Ulnar head absent	Beneath the humeral head	14%	Fewer potential entrapment sites; possible compression by hypertrophic humeral head

**Table 2:** Neurovascular entrapment potential of pronator teres muscle types.

Type	Median Nerve Relation to PT Heads	Typical Compression Site/Mechanism	Relative Entrapment Potential	Key Literature
**I—Between heads**	Median nerve passes **between humeral and ulnar heads**	Narrowing of **inter-head interval** by hypertrophy or fibrous bands; dynamic compression during resisted pronation [1,2,4,8,16].	**Moderate to high**—most frequently reported configuration with entrapment.	[1,2,4,8,16]
**II—Beneath both heads**	Nerve runs **deep to both heads**, forming a muscular tunnel	**Double-roofed canal** increases static pressure; both heads may require decompression when symptoms persist [2,8,9].	**High**—greatest risk due to confined passage.	[2,8,9]
**III—Beneath humeral head only**	Ulnar head absent; nerve passes **under humeral head alone**	Compression by hypertrophic humeral fibres or fibrous septa; subtle or position-dependent [8,9,10,18].	**Low to moderate**—less frequent but clinically relevant.	

**Table 3:** Reported observations on pronator teres (PT) morphological types and their relative entrapment potential.

Type	Summary of Reported Observations	Relative Entrapment Potential
Type I	Most frequently reported configuration associated with pronator syndrome. May mimic carpal tunnel syndrome if proximal symptoms are overlooked.	Moderate to high
Type II	Carries a higher risk of pronounced entrapment due to the double-headed tunnel. Surgical decompression of both heads often required in refractory cases.	High
Type III	Rarely reported but relevant in minimally invasive approaches; entrapment observed beneath the humeral head alone.	Low to moderate
General note	Awareness of anatomical variants is essential for correct diagnosis and targeted surgical planning.	—

**Table 4:** Imaging features and diagnostic pitfalls of pronator teres muscle types. Compiled summary of previously published imaging findings describing median nerve (MN) appearance and diagnostic challenges across PT types.

Type	Ultrasound (US)	Magnetic Resonance Imaging (MRI)	Diagnostic Pitfalls
I	MN visible within intermuscular cleft between humeral and ulnar heads; calibre changes reported; dynamic US considered helpful	T2-weighted and fat-suppressed sequences described focal thickening or hyperintensity at the site of entrapment	Frequently misinterpreted as carpal tunnel syndrome when only distal imaging is obtained
II	Limited visualisation due to deeper MN course beneath both heads; dynamic US during resisted pronation reported to improve detection	Often produces ambiguous or nonspecific signal changes; coronal and sagittal planes less reliable than axial	Entrapment may be overlooked without dynamic imaging and electrodiagnostic correlation
III	No typical intermuscular cleft; MN lies beneath humeral head only; subtle calibre variation may be visible	Advanced sequences (STIR, MR neurography) reported to detect intraneural oedema and subtle signal alterations	May create false impression of reduced entrapment risk due to absence of ulnar head landmark

**Table 5:** Clinical Imaging Tips—Literature-Based Insights for Diagnosing Pronator Syndrome.

Aspect	Key Findings and Practical Notes	Clinical Relevance
Imaging extent	Extending imaging proximal to the wrist improves detection of median nerve entrapment that may otherwise mimic CTS when only distal segments are assessed.	Prevents false CTS diagnosis.
Dynamic ultrasound	Dynamic US during resisted pronation increases sensitivity, especially in Type II configurations.	Enhances real-time entrapment detection.
MRI / MR neurography	In Type III variants, STIR or MRN sequences reveal subtle intraneural oedema or signal changes.	Supports early recognition of proximal compression.
Common pitfalls	Isolated distal imaging may yield a false CTS impression when proximal compression exists.	Avoids mislocalisation of pathology.
Multimodal evaluation	Combining US, MRI/MRN, and EDX is recommended for equivocal or complex cases.	Provides comprehensive diagnostic accuracy.

**Table 6:** Clinical implications and management strategies by pronator teres muscle type.

Type	Median Nerve Relationship	Typical Management Approach	Surgical Considerations/Reported Outcomes	Key Literature
**I—Between humeral and ulnar heads**	Nerve passes **between both heads**	**Conservative therapy**(physiotherapy, activity modification, US-guided perineural injections) as first-line [13,21,25].	Surgery indicated for persistent symptoms; **complete release of fibrous bands** yields favourable outcomes. Must differentiate from **lacertus syndrome** and **CTS** [4,9,10,12,26].	[4,9,10,12,13,21,25,26]
**II—Beneath both heads**	Nerve runs **deep to both heads**, forming a tunnel	Conservative care often **less effective** [3,8,9].	**Decompression of both heads** and excision of fibrous tissue recommended; technically demanding, with **higher recurrence** if incomplete [3,8,9].	[3,8,9]
**III—Beneath humeral head only**	Nerve passes **under humeral head**; ulnar head absent	Usually responsive to **conservative management** [9].	Surgery reserved for hypertrophic or fibrotic HH; **absence of UH complicates orientation**, making **pre-operative imaging** crucial [9,12].	

**Table 7:** Surgical access and procedural considerations by pronator teres muscle type.

Type	Recommended Surgical Approach	Advantages	Main Technical Challenges/Risks	Key Literature
**I—Between humeral and ulnar heads**	**Standard anterior exposure** via elbow flexor crease	Direct visualisation of both PT heads and intermuscular cleft; straightforward decompression [1,4].	Risk of **incomplete release** if fibrous bands not fully inspected; care to avoid nerve traction [10].	[1,4,10]
**II—Beneath both heads**	**Extended anterior approach** with deep dissection beneath both heads	Allows access to entire intramuscular tunnel and associated fibrous arches [8,9].	**Deep field**, proximity to **vascular branches** near ulnar head; incomplete decompression may cause recurrence [10,12].	[8,9,10,12]
**III—Beneath humeral head only**	**Focused anterior exposure** directed to humeral head; guided by preoperative imaging	Shorter exposure; suitable for **minimally invasive or endoscopic** access when anatomy is well defined [7,9,18].	**Loss of ulnar head landmark** complicates orientation; higher risk of **iatrogenic injury** if nerve course uncertain [10].	

## Data Availability

Not applicable. No new data were created or analyzed in this study. Data sharing is not applicable to this article.
